# Predictive Value of Fissured Tongue in Functional Dyspepsia Combined with Depression

**DOI:** 10.1155/2019/4596560

**Published:** 2019-06-17

**Authors:** Jizhong Song, Qiaomin Wang, Xuemei Xu, Chaolan Lv

**Affiliations:** The First Affiliated Hospital of USTC, Division of Life Sciences and Medicine, University of Science and Technology of China, No. 17 Lujiang Road, Hefei, Anhui, 230001, China

## Abstract

Anxiety and depression are common in functional dyspepsia (FD) patients. Although fissured tongue (FT) is often observed in FD, its clinical value in such patients is rarely reported. We analyzed clinical data of FD patients with FT with the aim of elucidating the clinical value of FT in FD. This study suggests FD patients with different types of FT with the course of disease and the 9-item Patient Health Questionnaire (PHQ9) showed a significant difference. The PHQ9, course of disease, and self-rated dyspepsia symptoms (SRDS) correlated positively with the types of FT by the Spearman rank analysis. Epigastric pain, bloating, nausea, and SRDS showed a significant difference between FT-FD and nonfissured tongue- (NFT-) FD as well as between FD patients with and without symptoms of depression. Many FD patients also have FT, which may be associated with depressive symptoms. The longer the course of disease, the more serious the fissured tongue; thus, it may provide a predictive value for the diagnosis of depressive symptoms in FD patients.

## 1. Introduction

Functional dyspepsia (FD) is the most common gastrointestinal disease, accounting for the prevalence of more than 20% [1]. The symptoms of some FD patients are relapsing and remitting, which can seriously affect their quality of life. Clinically, some FD patients have psychiatric symptoms such as anxiety and depression [2], which are reported as common causes of severity in FD [3]. However, it remains difficult to early uncover mental health symptoms of FD patients. Although all kinds of mental health symptom questionnaires are now widely used, such as the Hospital Anxiety and Depression scale [4], there remain many deficiencies in the clinical application of these questionnaires, particularly regarding their speed and accuracy in identifying whether FD outpatients may have psychological problems.

Fissured tongue (lingua plicata) can be distinguished from geographic or map tongue (erythema migrans). Fissured tongue is believed to be related to aging [5]. *Tongue diagnosis* has a long history in China and is one of the most important diagnostic means of traditional Chinese medicine (TCM). According to TCM, fissured tongue is related to impairment of *yin* by excessive heat. The theory of *tongue diagnosis* in TCM is very complex and difficult to understand among non-TCM doctors. In the clinical setting, we find many patients with fissured tongue, although it is difficult to judge the relationship between fissured tongue and specific diseases by analyzing the available literature. At present, although extensive literature has reported that there may be some relationship between fissured tongue and psoriasis [6], in fact, a substantial number of FD patients have also been discovered to have fissured tongue. Therefore, we analyzed the clinical data of FD patients with fissured tongue and explored the clinical significance of this relationship.

## 2. Methods

### 2.1. Patients and Procedures

FD patients were selected from the outpatient registry of the Gastroenterology Department of Anhui Provincial Hospital (i.e., The First Affiliated Hospital of USTC, Division of Life Sciences and Medicine, University of Science and Technology of China) from December 2016 to July 2017. FD was diagnosed according to the Rome IV criteria [7]. According to the voluntary principle, the patients were required to complete the 9-item Patient Health Questionnaire (PHQ9) and 7-item Generalized Anxiety Disorder Scale (GAD7) and to take pictures of their tongues. All patients underwent physical examination, detailed history collection, and gastroscopy to rule out organic diseases. Informed consent was obtained after fully understanding the benefits and risks. After removing patients according to the exclusion criteria, 95 FD patients were enrolled in the study, 20 of whom without fissured tongue constituted the control group. This study was approved by the Anhui Provincial Hospital Medical Research Ethics Committees (P-021).

### 2.2. Fissured Tongue Classification

**Figure 1:**
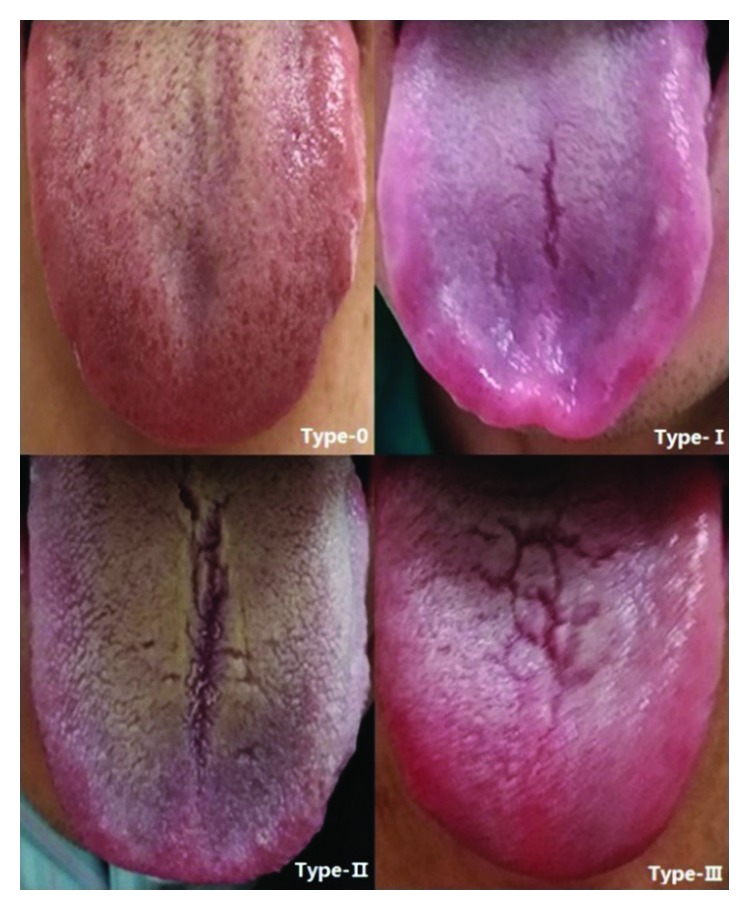
Fissured tongue classification: Type 0, no groove; Type I, single small groove, less than half the length of the surface of the tongue, with no forks or short forks; Type II, single, thicker, deeper groove, longer than half the length of the surface of the tongue, with or without forks; and Type III, two or more grooves, deep and shallow, with irregular arrangement.

Fissured tongue is diagnosed clinically on the basis of fissures on the dorsal surface of the tongue with at least one groove and smooth-surfaced papillae of varying sizes observed in the region of fissure [8]. According to the length and shape of the groove, it is divided into four types: Type 0, no groove; Type I, single small groove, less than half the length of the surface of the tongue, with no forks or short forks; Type II, single, thicker, deeper groove, longer than half the length of the surface of the tongue, with or without forks; and Type III, two or more grooves, deep and shallow, with irregular arrangement (Figure 1). FD patients with fissured tongue were designated as FT-FD and those without fissured tongue as NFT-FD.

### 2.3. Exclusion Criteria

Exclusion criteria were (1) skin and rheumatic diseases such as psoriasis and lupus erythematosus; (2) combined with dental caries and other oral diseases; (3) peptic ulcer, gastric cancer, or other organic dyspepsia; (4) Down syndrome; (5) and obvious improvement in tongue fissure after taking vitamin B compounds for 4 weeks.

### 2.4. General Information and Questionnaire

General information on age, sex, height, weight, and course of disease of all FD patients was documented. Patients compiled self-rated dyspepsia symptoms [9], the total scores of which were designated SRDS. Regarding the self-administered PHQ9 and GAD7. The cutoff PHQ9 score was 5, 5 points or more equate to FD patients with depression symptoms (DP-FD), and less than 5 points equate to nondepressed FD patients (NDP-FD) [10]. The cutoff GAD7 score was 5, 5 points or more equates to FD patients with anxiety symptoms, and less than 5 points equate to nonanxiety FD patients [11].

The authors have no ethical conflicts to disclose.

### 2.5. Statistical Analysis

SPSS 20 statistical software was used for statistical analysis. The measurement data of normal distribution are expressed as the mean ± SD. Independent Student's *t*-test was used in the two-group comparison, and variance analysis was used to compare the three groups. The numeric data were expressed in percentages and the chi-squared test. The Spearman rank correlation test was used to analyze the correlation of types of fissured tongue. *P* values of <0.05 were considered statistically significant.

## 3. Results

### 3.1. General Information

A total of 95 FD patients were enrolled in the study. There were 44 men (oldest 80 years, youngest 18 years, and median 48.5 years) and 51 women (oldest 73 years, youngest 24 years, and median 51 years). There were 45 cases (45/95, 47.37%) of epigastric pain syndrome (EPS), 34 cases (34/95, 35.79%) of postprandial discomfort syndrome (PDS), and 16 cases (16/95, 16.84%) of overlapping syndrome. There were 75 cases (75/95, 78.95%) of fissured tongue (FT-FD; 34 men and 41 women, *χ*^2^ = 1.307, *P* = 0.253) and 20 cases (20/95, 21.05%) of nonfissured tongue (NFT-FD; 10 men and 10 women). The PHQ9 scored >5 points in 70 cases (70/95, 73.68%), and the GAD7 scored >5 points in 66 cases (66/95, 69.47%).

### 3.2. Comparison of Self-Rated Dyspepsia Symptom Scores between FT-FD and NFT-FD Patients

The comparison of self-rated dyspepsia symptom scores between FT-FD and NFT-FD revealed that epigastric pain (1.813 ± 0.711 and 1.400 ± .681, *t* = −2.331, and *P* = 0.022), bloating (1.720 ± .938 and 1.150 ± 0.671, *t* = −2.545, and *P* = 0.013), nausea (1.387 ± 0.820 and 0.550 ± 0.759, *t* = −4.114, and *P* = 0.001), and SRDS (10.107 ± 2.311 and 8.750 ± 1.293, *t* = −2.516, and *P* = 0.014) showed a significant difference. Postprandial fullness, early fullness, vomiting, belching, and epigastric burning showed no statistical significance between the groups (Table 1).

### 3.3. Comparison of Self-Rated Dyspepsia Symptom Scores between Patients with DP-FD and NDP-FD

The comparison of self-rated dyspepsia symptom scores between patients with DP-FD and NDP-FD revealed that epigastric pain (1.886 ± 0.671 and 1.280 ± 0.678, *t* = −3.863, and *P* = 0.001), bloating (1.800 ± 0.910 and 1.040 ± 0.676, *t* = −4.380, and *P* = 0.001), nausea (1.429 ± 0.827 and 0.600 ± 0.707, *t* = −4.459, and *P* = 0.001), and SRDS (10.400 ± 2.095 and 8.200 ± 1.633, *t* = 4.755, and *P* = 0.001) showed a significant difference between DP-FD and NDP-FD patients, whereas postprandial fullness, early fullness, vomiting, belching, and epigastric burning were not significant (Table 2).

### 3.4. Comparison of Different Types of Fissured Tongue

Regarding the comparison of different types of fissured tongue, there was a significant difference between men and women (*χ*^2^ = 236.906, *P* = 0.001). The course of disease and PHQ9 were also significantly different (7.300 ± 1.780, 9.000 ± 2.890, 10.585 ± 4.342, and 13.563 ± 5.006; *F* = 8.654; and *P* = 0.001) and (4.300 ± 2.430, 9.000 ± 1.749, 8.195 ± 3.586, and 10.000 ± 2.944; *F* = 13.304; and *P* = 0.001) of different types of fissured tongues. There was no statistical significance in age, body mass index (BMI), GDA7 score, and SRDS score (Table 3).

### 3.5. Correlation Analysis of Types of Fissured Tongue

The analysis of the correlation between types of fissured tongue and PHQ9, GDA7, and other parameters in FD patients was conducted by the Spearman rank analysis. The PHQ9 (odds ratio (OR) = 0.461, *P* = 0.001), course of disease (OR = 0.456, *P* = 0.001), and SRDS (OR = 0.275, *P* = 0.007) were positively correlated with the types of fissured tongue, but the BMI (OR = −0.409, *P* = 0.001) was negatively correlated. There was no correlation with age and GAD7 (Table 4).

## 4. Discussion

Fissured tongue, which is diagnosed by the presence of at least one groove and smooth-surfaced papillae of varying sizes seen in the region of the tongue fissure, with no inflammatory infiltration, is readily found in the clinical setting, in contrast to geographic or map tongue. The description of fissured tongue in TCM has a long history. TCM is usually dialectically integrated with tongue coating and color [12], which is clinically difficult for non-TCM doctors to understand and apply. Fissured tongue is not uncommon in the Chinese population, with a detection rate of about 3.15% [13]. Many scholars around the world have begun to pay attention to fissured tongue in an attempt to explore the relationship between fissured tongue and disease (and health) [14]. Feil and Filippi [5] conducted a clinical study of 1000 patients with fissured tongue and found that the incidence among middle-aged people was significantly higher, with smoking possibly a major risk factor. The present study showed that there was no significant difference between men and women in the proportion of fissured tongue, and the numbers of Type II and III fissured tongue is more in women than in men, but women in Hefei hardly smoked. As early as 1984, Hietanen et al. [15] found fissured tongue in about 9.5% of 200 psoriasis patients. Since then, there have been many reports stating that fissured tongue is related to psoriasis [15, 16], although the pathological difference between fissured tongue and psoriasis is difficult to ascertain [6]. Many case reports of fissured tongue have also appeared [17, 18]. Therefore, the clinical value of fissured tongue is still worthy of a further study.

Modern computer technology provides new research tools for disease diagnosis, helping many researchers to study the relationship between tongue manifestations and diseases. Gareus et al. [19] used digital camera technology to photograph standardized tongue images to investigate the diagnostic approach to tongue pathology. Qi et al. [20] set up a TDA-1 tongue-imaging device to take photographs for the premise of standardized tongue acquisition and color reproduction, making feasible the preliminary objectification of tongue color classification in TCM. Wu et al. [21] established the automatic tongue diagnosis system (ATDS), which noninvasively captures tongue images and can provide objective and reliable diagnostic information for the diagnosis of digestive system diseases. These are but examples of the continued scientific search for data with which to explore the value of tongue imaging in the clinical diagnosis of diseases. Manifestations on the tongue are complex, and the relationship between tongue manifestations and disease has yet to be determined. The present study of FD patients found a positive correlation between fissured tongue and PHQ9; moreover, there are significant differences in PHQ9 between different types of fissured tongue. Thus, noninvasive and standardized tongue diagnosis should be further explored [22].

FD has a relatively high incidence, affecting about 10% of the population in the Commonwealth countries [23], whereas in China a considerably higher incidence has been estimated, namely, about 23.5% [24]. FD is a cause of symptoms that cannot be explained by routine clinical evaluation. However, in recent years, FD has been found to have structural abnormalities [25]. These findings challenge the classical paradigm that patients with FD show no structural changes in the gastrointestinal tract; for example, Vanheel et al. [26] found that impaired intestinal barrier function is a pathophysiological mechanism in FD. A further study of 24 FD patients demonstrated ultrastructural changes in the degranulation state of eosinophils and mast cells, suggesting that eosinophil and mast cell activation play a role in the pathophysiology of FD [27]. Using magnetic resonance brain imaging technology in 12 patients with high-fat dyspepsia, it was found that the frontal middle gyrus, insula, and occipital cortex were activated in FD patients [28]. Therefore, the Rome IV standard holds that FD patients have abnormal brain-gut interaction [7]. The present study shows that there are significant differences in BMI among FD patients with different types of fissured tongue, with BMI decreasing along with the increase in the grade of fissured tongue, thus suggesting that FD patients with fissured tongue have abnormal metabolism.

The treatment of FD is not always easy. Many patients have severe symptoms and relapse, which seriously affect their quality of life and lead to refractory FD. This group of FD patients is more commonly associated with mental disorders such as anxiety and depression. These psychological symptoms will aggravate the symptoms of FD, resulting in symptom relapse and refractory disease. The causal link between depression or anxiety and FD is still controversial. At present, the general view is that FD and anxiety/depression are comorbid [29]. In the present study, the PHQ9 in 73.68% of FD patients was >5 points and the GAD7 score in 69.47% was more than >5 points, suggesting that anxiety and depression are more common in FD patients [30]. Using a PHQ9 score of 5 as a cutoff value, FD patients were divided into the DP-FD and NDP-FD groups. Epigastric pain, bloating, nausea, and SRDS were significantly higher in DP-FD than in NDP-FD patients. Depression is characterized by marked and persistent mood depression, without obvious organic and histological changes. However, in recent years, studies have found that depression also produces inflammation, and some scholars even consider depression an inflammatory disease [31]. Using resting-state functional magnetic resonance imaging, Oathes et al. [32] found that a single conceptual model alone provides an incomplete mapping of psychopathology to neurobiology in patients with anxiety and depression. Yuen et al. [33], in their research on elderly patients with depression, found that structural abnormalities of the posterior subgenual cingulate gyrus and uncinate fasciculus may perpetuate apathetic states by interfering with prefrontal cortical recruitment of limbic activity essential to motivational behavior. Therefore, FT-FD patients may have an abnormal organ structure, such as anatomical changes in the tongue. Therefore, by observing the sign of fissured tongue, we may quickly identify whether FD patients have depressive symptoms in the early stage and provide psychotherapy.

The present study found that FD patients were more likely to have fissured tongue. After supplementation with a large number of vitamin B compounds, there was no significant improvement in fissured tongue after 1 month. Available data do not suggest that fissured tongue is related to vitamin B deficiency caused by malnutrition in FD patients [34]. On dividing FD patients into FT-FD and NFT-FD we also found that epigastric pain, bloating, nausea, and SRDS were significantly different, similar to results in DP-FD patients. Analysis of available data has revealed that fissured tongue is associated with autoimmune disorders such as psoriasis, and that these diseases often have an inflammatory response. Although FD and depression show no routine biochemical and histological changes, more evidence supports the existence of low grades of inflammatory response [26, 27, 31], as well as structural and functional changes in the central nervous system [35, 36]. Therefore, fissured tongue in FD patients with depression may have a specific pathophysiological basis. Fissured tongue can also occur in psoriasis and other diseases, with a total incidence of about 8%–30% [37, 38]. In fact these diseases are readily associated with mental health symptoms such as depression [39]. To date no literature has reported on whether fissured tongue in patients is associated with depressive symptoms. Taken together, we consider the results of the present study to suggest that the presence of fissured tongue is related to depressive symptoms in FD patients.

## 5. Conclusion

We found that fissured tongue was frequently observed in FD patients, especially in those with depressive symptoms. Through the analysis of the existing research data combined with the results of this study, we conclude that the presence of fissured tongue may be related to depressive symptoms, and that this will represent a predictive value for the diagnosis of depressive symptoms in FD patients.

## Figures and Tables

**Table 1:** Comparison of self-rated dyspepsia symptom scores between FT-FD and NFT-FD patients.

	FT-FD	NFT-FD	*t* value	*P* value
Epigastric pain	1.813 ± 0.711	1.400 ± .681	-2.331	0.022∗
Bloating	1.720 ± .938	1.150 ± 0.671	-2.545	0.013∗
Postprandial fullness	1.600 ± 0.788	1.700 ± 0.571	0.530	0.597
Early fullness	1.093 ± 1.187	1.200 ± 0.834	0.377	0.707
Nausea	1.387 ± 0.820	0.550 ± 0.759	-4.114	0.001∗∗
Vomiting	0.120 ± 0.327	0.050 ± 0.224	-0.901	0.370
Belching	1.387 ± 0.837	1.400 ± 0.754	0.065	0.949
Epigastric burning	0.987 ± 0.908	1.300 ± 0.801	1.404	0.164
SRDS	10.107 ± 2.311	8.750 ± 1.293	-2.516	0.014∗

∗ represent *P* value less than 0.05. ∗∗ represent *P* value less than 0.01. SRDS: total scores of self-rated dyspepsia symptoms. Epigastric pain (1.813 ± 0.711 and 1.400 ± .681, *t* = −2.331, and *P* = 0.022), bloating (1.720 ± .938 and 1.150 ± 0.671, *t* = −2.545, and *P* = 0.013), nausea (1.387 ± 0.820 and 0.550 ± 0.759, *t* = −4.114, and *P* = 0.001), and SRDS (10.107 ± 2.311 and 8.750 ± 1.293, *t* = −2.516, and *P* = 0.014) were significantly different between FT-FD and NFT-FD patients; postprandial fullness, early fullness, vomiting, belching, and epigastric burning were not significant.

**Table 2:** Comparison of self-rated dyspepsia symptom scores between DP-FD and NDP-FD patients.

	DP-FD	NDP-FD	*t* value	*P* value
Epigastric pain	1.886 ± 0.671	1.280 ± 0.678	-3.863	0.001∗∗
Bloating	1.800 ± 0.910	1.040 ± 0.676	-4.380	0.001∗∗
Postprandial fullness	1.643 ± 0.799	1.560 ± 0.583	-0.475	0.636
Early fullness	1.157 ± 1.211	1.000 ± 0.817	-0.601	0.550
Nausea	1.429 ± 0.827	0.600 ± 0.707	-4.459	0.001∗∗
Vomiting	0.129 ± 0.337	0.040 ± 0.200	-1.560	0.123
Belching	1.386 ± 0.856	1.400 ± 0.707	0.075	0.941
Epigastric burning	0.971 ± 0.900	1.280 ± 0.843	1.495	0.138
SRDS	10.400 ± 2.095	8.200 ± 1.633	-4.755	0.001∗∗

∗∗ represent *P* value less than 0.01. SRDS: total scores of self-rated dyspepsia symptoms. Epigastric pain (1.886 ± 0.671 and 1.280 ± 0.678, *t* = −3.863, and *P* = 0.001), bloating (1.800 ± 0.910 and 1.040 ± 0.676, *t* = −4.380, and *P* = 0.001), nausea (1.429 ± 0.827 and 0.600 ± 0.707, *t* = −4.459, and *P* = 0.001), and SRDS (10.400 ± 2.095 and 8.200 ± 1.633, *t* = 4.755, and *P* = 0.001) were significantly different between DP-FD and NDP-FD patients; postprandial fullness, early fullness, vomiting, belching, and epigastric burning were not significant.

**Table 3:** Comparison of different types of fissured tongues.

	Type 0	Type I	Type II	Type III		
					*χ* ^2^ value	*P* value
Man	11	10	16	7		
Woman	9	8	25	9	236.906	0.001∗∗
					*F* value	*P* value
Age	52.000 ± 13.171	47.444 ± 11.808	49.073 ± 11.073	51.812 ± 15.833	0.599	0.617
Course of disease	7.300 ± 1.780	9.000 ± 2.890	10.585 ± 4.342	13.563 ± 5.006	8.654	0.001∗∗
BMI	21.290 ± 2.299	20.617 ± 2.6336	20.120 ± 2.898	19.557 ± 2.517	1.457	0.232
PHQ9	4.300 ± 2.430	9.000 ± 1.749	8.195 ± 3.586	10.000 ± 2.944	13.304	0.001∗∗
GDA7	6.800 ± 2.726	6.944 ± 3.556	7.195 ± 3.265	5.938 ± 3.276	0.592	0.622
SRDS	8.750 ± 1.292	9.778 ± 1.665	10.073 ± 2.544	10.563 ± 2.366	2.474	0.067

∗∗ represent *P* value less than 0.01. BMI: body mass index; PHQ9: 9-item Patient Health Questionnaire; GAD7: 7-item Generalized Anxiety Disorder Scale; SRDS: total scores of self-rated dyspepsia symptoms. The course of disease and PHQ9 were significantly different (7.300 ± 1.780, 9.000 ± 2.890, 10.585 ± 4.342, and 13.563 ± 5.006; *F* = 8.654; and *P* = 0.001) and (4.300 ± 2.430, 9.000 ± 1.749, 8.195 ± 3.586, and 10.000 ± 2.944; *F* = 13.304; and *P* = 0.001) of different types of fissured tongues. There was no statistical significance in age, BMI, GDA7, and SRDS.

**Table 4:** Correlation analysis of types of fissured tongue.

	OR	*P* value
Age	0.025	0.812
PHQ9	0.461	0.001∗∗
GAD7	-0.072	0.486
MBI	-0.409	0.001∗∗
Course of disease	0.456	0.001∗∗
SRDS	0.275	0.007∗∗

∗∗ represent *P* value less than 0.01. BMI: body mass index; PHQ9: 9-item Patient Health Questionnaire; GAD7: 7-item Generalized Anxiety Disorder Scale; SRDS: total scores of self-rated dyspepsia symptoms. The PHQ9 (OR = 0.461, *P* = 0.001), course of disease (OR = 0.456, *P* = 0.001), and SRDS (OR = 0.275, *P* = 0.007) showed a positive correlation with types of fissured tongue, but the BMI (OR = −0.409, *P* = 0.001) was negatively correlated. No correlation was shown with age and GAD7.

## Data Availability

No data were used to support this study.

## Additional Points

*Provenance and Peer Review.* This study is not commissioned and is externally peer reviewed.
